# Changes and Clinical Significance of PIVKA-II in Hepatitis E Patients

**DOI:** 10.3389/fpubh.2021.784718

**Published:** 2022-01-25

**Authors:** Youran Chen, Yanyan Yang, Shanshan Li, Minghao Lin, Xueting Xie, Huifang Shi, Yuchun Jiang, Sijie Zheng, Hui Shao, Naibin Yang, Mingqin Lu

**Affiliations:** ^1^Department of Infectious Diseases, The First Affiliated Hospital of Wenzhou Medical University, Wenzhou, China; ^2^Department of Infectious Diseases, Taizhou Hospital of Zhejiang Province, Taizhou, China; ^3^Department of Infectious Diseases, Ningbo First Hospital, Ningbo, China

**Keywords:** hepatitis E, PIVKA-II, liver function, liver insufficiency, acute hepatitis

## Abstract

Increased protein induced by vitamin K absence or antagonist-II (PIVKA-II) levels had been widely reported in patients with hepatocellular carcinoma (HCC) and chronic hepatitis. However, the role of PIVKA-II in hepatitis E is unclear. The aim of this study was to clarify the changes related with PIVKA-II and its clinical significance in hepatitis E. We enrolled 84 patients with hepatitis E hospitalized in two hospitals from December 2019 to June 2021. The levels of serum PIVKA-II and related serological indicators in the patients were determined to elucidate the role of PIVKA-II in hepatitis E. We observed that 59.51% (50/84) of patients showed an increase in PIVKA-II levels. Compared with the normal PIVKA-II group (<32 mAU/L), patients in the elevated PIVKA-II group (>32 mAU/L) had much higher serum total bilirubin (TBIL), direct bilirubin (DBIL), indirect bilirubin (IBIL), and total bile acid (TBA) levels (*p* < 0.05 for each). Compared with the slightly elevated PIVKA-II group (32–125 mAU/L), patients in the significantly elevated PIVKA-II group (>125 mAU/L) had much lower serum albumin, alanine aminotransferase (ALT), aspartate transaminase (AST) levels, and longer days for the hospital stay (*p* < 0.05 for each). The association of PIVKA-II with TBIL and DBIL was an inverted U-shaped curve with an inflection point at 199.1 mAU/L). The association of PIVKA-II with IBIL was a U-shaped curve with an inflection point at 18.6 mAU/L while the association of PIVKA-II with albumin was an inverted U-shaped curve with an inflection point at 18.6 mAU/L. With the improvement of the disease, PIVKA-II levels were gradually decreased and finally returned to normal. This trend was consistent with that of bilirubin, and a peak appeared in the third week. Therefore, findings from our study show that the increase in PIVKA-II levels can be related to the degree of hepatic insufficiency in patients with hepatitis E, wherein PIVKA-II levels are transiently increased, and the trend of change can be related to the disease course.

## Introduction

Hepatitis E is an acute self-limiting disease caused by the hepatitis E virus (HEV) and is mainly transmitted through the fecal-oral route (1). According to statistics from the WHO, approximately 20 million people worldwide are infected with HEV each year, with more than 3 million cases of acute hepatitis E and 70,000 patient deaths (2). Thus, hepatitis E has become one of the most important public health problems worldwide.

Protein induced by vitamin K absence or antagonist-II (PIVKA-II), or des-gamma-carboxy prothrombin (DCP), is produced when the body is deficient in vitamin K and prothrombin cannot be shuttled into prothrombin or prothrombin is not fully shuttled, increasing the prothrombin levels and, consequently, PIVKA-II levels (3, 4). PIVKA-II is often used for the auxiliary detection of patients with hepatocellular carcinoma (HCC) accompanied with Alpha-fetoprotein (AFP) (5). Large scales of studies had analyzed PIVKA-II for patients with HCC using patients with chronic hepatitis as the control group and found slightly elevated PIVKA-II in patients with chronic hepatitis (6). We speculated that there might also be an increase of PIVKA-II in acute hepatitis, and the increase might be related to the degree of liver damage. Moreover, the role of PIVKA-II in hepatitis E is still unclear. Thus, we aimed to explore the relationship between PIVKA-II levels and HEV infection in this study, to obtain a better understanding of the role of PIVKA-II in hepatitis E.

## Materials and Methods

### Patients

We enrolled 84 patients with hepatitis E in this study, including 69 patients who were referred to the First Affiliated Hospital of Wenzhou Medical University from December 2019 to June 2021 and 15 patients who were admitted to Taizhou Hospital of Zhejiang Province from December 2019 to June 2021. Inclusion criteria were as followed: HEV infection was diagnosed by testing for anti-HEV immunoglobulin M (IgM) using a serum enzyme-linked immunosorbent assay (ELISA) test. Hepatitis E cases were defined based on positive serum anti-HEV IgM, combined with clinical presentation of acute hepatitis (e.g., elevated liver enzymes and/or jaundice and/or non-specific symptoms, such as fatigue, itching, and nausea). The following exclusion criteria were established as previously reported (7): (1) use of antibiotics during the previous month; (2) current bacterial or fungal infections; (3) co-infection with hepatitis A virus, hepatitis B virus, or hepatitis C virus, or the presence of alcoholic fatty liver disease; (4) drug-induced liver disease; (5) autoimmune liver disease; (6) liver cancer, reproductive embryonic cancer, and/or female pregnancy; (7) co-infection with cytomegalovirus or Epstein–Barr virus; (8) presence of metabolic associated fatty liver disease; (9) approval for liver transplantation; and (10) incomplete data.

Studies involving human participants were reviewed and approved by the ethics committee of the First Affiliated Hospital of Wenzhou Medical University and Taizhou Hospital of Zhejiang Province (approval number: no. 2021-zz-162). Due to the retrospective study design involving electronic health records and no additional interventions, written informed consent was waived from the patients or their relatives.

### Measurement of the PIVKA-II and Other Serological Indicator Levels

Serum levels of PIVKA-II were detected using an Abbott I 1000 automatic immunoassay analyzer. Peripheral blood was obtained from each patient, no matter fasting or not. The serum was obtained by centrifuging for 5 min at 3,000 rpm and stored at −80°C until testing. All serum samples were kept in duplicate. All the operational processes regarding the measurement of PIVKA-II were blind to measurers. Detection of HEV IgM *via* ELISA was performed by Shanghai Kehua Biological Engineering Co., Ltd. Alanine aminotransferase (ALT), aspartate transaminase (AST), alkaline phosphatase (ALP), γ-glutamyl transpeptidase (GGT), total bilirubin (TBIL), direct bilirubin (DBIL), indirect bilirubin (IBIL), total bile acid (TBA), and albumin levels were analyzed using a Beckman AU5800 automatic biochemical detector. AFP was analyzed using a Beckman DXI800. The prothrombin time (PT) was analyzed using the Stago R Max. The above reagents were all original kits, and the standard operating procedure was strictly followed during the test.

### Statistical Analysis

All statistical analyses were performed using SPSS version 26.0, EmpowerStats (http://www. empowerstats.com), and package R (http://www.Rproject.org). For continuous data, the data were displayed as median (minimum–maximum) or as the actual value of the classification data. Number (%) was for categorical variables. Baseline features were summarized using descriptive statistics. Groups were compared using chi-square tests for categorical variables, Mann-Whitney U tests were used for continuous variables for comparing two independent groups. A *p* < 0.05 was considered to be statistically different.

## Results

### Baseline Characteristics Based on Normal or Elevated PIVKA-II

**Figure 1:**
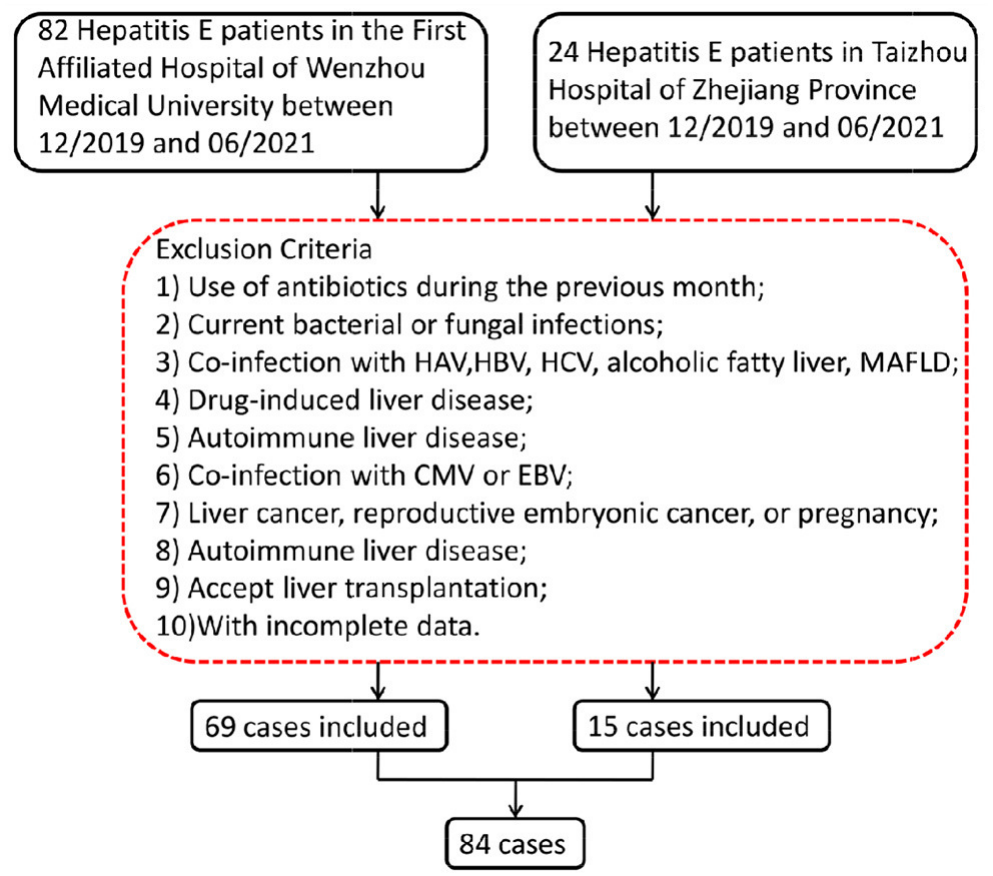
Screening flow chart for patients with hepatitis E. HAV, hepatitis A virus; HBV, hepatitis B virus; HCV, hepatitis C virus; MAFLD, metabolic associated fatty liver disease; CMV, cytomegalovirus; EBV, Epstein–Barr virus.

**Figure 2:**
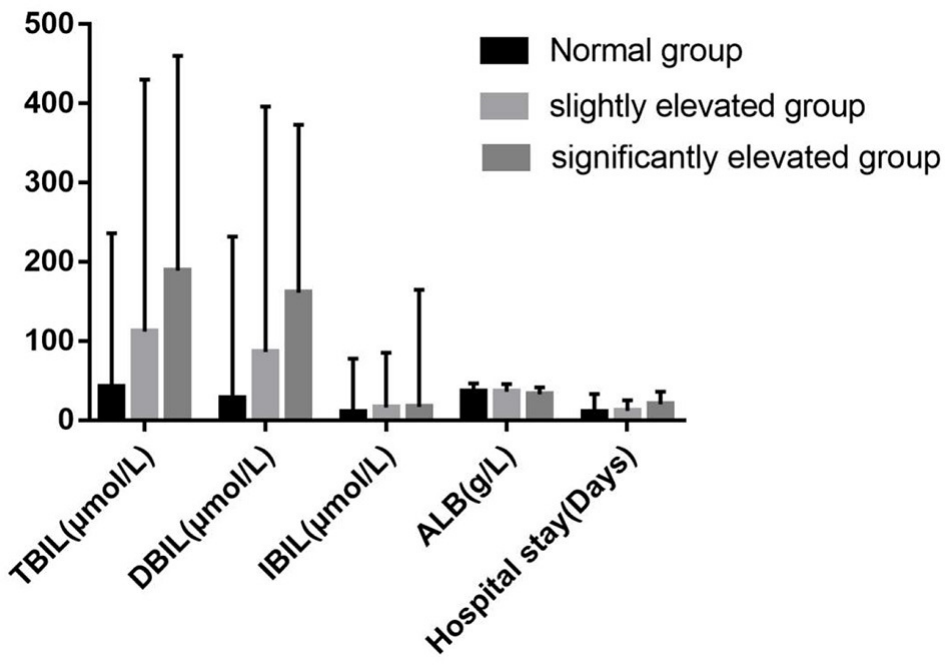
Levels of clinical biochemical indexes in different groups of serum PIVKA-II levels. PIVKA, protein induced by vitamin K absence or antagonist-II.

The flow chart for screening the patients with hepatitis E is shown in Figure 1. A total of 84 patients with hepatitis E aged 25–77 years were included in our study, with the baseline characteristics according to normal PIVKA-II or elevated PIVKA-II are presented in Table 1 and Figure 2. Of them, 67 were men and 17 were women. The median age was 53 years old, ranging from 25 to 77. Since the range of reference value for PIVKA-II is 11–32 mAU/L, we adopted 32 mAU/L as the cut-off value to divide the 84 patients into the normal group (n = 34) with PIVKA-II ≤ 32 mAU/L and elevated group (n = 50) with PIVKA-II >32 mAU/L. The median level of PIVKA-II in normal group was 24.6 mAU/L range from 10.6 to 31.4 mAU/L, while their counterpart in the elevated group was much higher as 53.8 mAU/L with significant statistical differences (*p* < 0.001). Compared with the normal PIVKA-II group, patients in the elevated PIVKA-II group had much higher serum TBIL, DBIL, IBIL, and TBA levels (*p* < 0.05 for each). There were no significant differences in the distribution of age and gender between the two groups. Moreover, no significant differences were found in levels of albumin, ALT, AST, ALP, GGT, AFP, PT, and days for the hospital stay.

**Table 1:** Baseline characteristics of patients with hepatitis E.

**Parameter**	**Total (*n* = 84)**	**Normal group (*n* = 34)**	**Elevated group (*n* = 50)**	***P* value**
Age (years)	53 (25–77)	52 (29–77)	52 (25–74)	0.616
Gender				0.084
Male	67 (79.8%)	24 (70.6%)	43 (86.0%)	
Female	17 (20.2%)	10 (29.4%)	7 (14.0%)	
TBIL (μmol/L)	115.5 (7–460.3)	42.0 (7.0–236.0)	120.5 (12.0–460.3)	<0.001
DBIL (μmol/L)	94.6 (2–396)	27.8 (2.0–232.0)	95.5 (6.0–396.0)	<0.001
IBIL (μmol/L)	21.0 (4–164.7)	10.6 (4.0–77.9)	16.0 (4.0–164.7)	0.015
Albumin (g/L)	35.8 (23.9–46.4)	36.2 (23.9–46.4)	35.5 (27.0–45.6)	0.512
ALT (U/L)	1,405.14 (25–5,406)	724.0 (25–5,406)	1,217.0 (32.0–4,228.0)	0.219
AST (U/L)	772.7 (22–6,436)	166.0 (22–6,436)	382.5 (33.0–5,720.0)	0.111
ALP (U/L)	208.8 (61–475)	174.0 (61.0–404.0)	201.0 (84.0–475.0)	0.155
GGT (U/L)	276.4 (31–903)	232.5 (34.0–640.0)	209.5 (31.0–903.0)	0.781
TBA (μmol/L)	168.1 (2–477)	104.1 (2.0–477.0)	238.5 (2.4–450.0)	0.039
AFP (ng/mL)	46.4 (1.22–474.0)	33.2 (1.2–340.1)	20.4 (1.5–474.0)	0.830
PT (s)	21.5 (10–337)	14.0 (12.2–337.0)	13.5 (10.0–19.8)	0.247
PIVIK-II (mAU/L)	66.7 (10.6–546.1)	24.6 (10.6–31.4)	53.8 (33.0–9,235.0)	<0.001
Hospital days	13.7 (4–36)	10.5 (4.0–33.0)	13.0 (7.0–36.0)	0.085

*Median (min–max) for continuous variables: the p was calculated by the linear regression model. N (%) for categorical variables: the p was calculated by the chi-square test. p < 0.05 was considered statistically significant. TBIL, total bilirubin; DBIL, direct bilirubin; IBIL, indirect bilirubin; TBA, total bile acid; ALT, alanine aminotransferase; AST, aspartate transaminase; GGT, γ-glutamyl transpeptidase; TBA, total bile acid; AFP, Alpha-fetoprotein*.

### Association Between Clinical Biochemical Indexes and Degree of Elevated PIVKA-II

Following Marrero (8), we used 125 mAU/ml as the grouping cut-off value. Further, we divided the above 50 patients with elevated PIVKA-II levels into two groups according to the degree of elevation: the slightly elevated group with PIVKA-II 33–125 mAU/ml and the significantly elevated group with PIVKA-II >125 mAU/L. The median level of PIVKA-II in the significantly elevated group was 318.1 mAU/ml, much higher than their counterpart of 48.8 mAU/ml in the slightly elevated group (*p* < 0.001). Compared with the slightly elevated PIVKA-II group, patients in the significantly elevated PIVKA-II group had much lower serum albumin, ALT, AST levels, and longer days for the hospital stay (*p* < 0.05 for each). However, there were no significant differences in the serum TBIL, DBIL, IBIL, ALP, GGT, and TBA levels between the two groups. The detailed information is shown in Table 2 and Figure 2.

**Table 2:** Association between clinical biochemical indexes and degree of elevated PIVKA-II.

**Parameter**	**slightly elevated (<125 mAU/L)**	**significantly elevated (>125 mAU/L)**	***P* value**
PIVKA-II (mAU/L)	48.8 (33.0–124.6)	318.1 (127.2–9,235.0)	<0.001
TBIL (μmol/L)	112.0 (12.0–430.0)	189.0 (45.0–460.3)	0.050
DBIL (μmol/L)	86.0 (6.0–396.0)	161.0 (37.0–373.0)	0.037
IBIL (μmol/L)	16.0 (4.0–85.3)	17.0 (8.0–164.7)	0.390
Albumin (g/L)	36.2 (27.6–45.6)	32.5 (27.0–41.3)	0.018
ALT (U/L)	1,461.0 (97.0–4,228.0)	237.0 (32.0–3,203.0)	0.002
AST (U/L)	564.0 (44.0–5,720.0)	89.0 (33.0–1,294.0)	0.008
ALP (U/L)	200.0 (84.0–466.0)	208.5 (115.0–475.0)	0.921
GGT (U/L)	219.0 (36.0–840.0)	150.0 (31.0–903.0)	0.181
TBA (μmol/L)	241.0 (2.4–450.0)	202.7 (17.0–264.0)	0.431
AFP (ng/mL)	15.9 (1.5–474.0)	28.4 (4.2–455.2)	0.383
PT (s)	13.4 (1.0–18.0)	15.0 (12.3–19.8)	0.165
Hospital stay (Days)	12.0 (7.0–25.0)	20.0 (10.0–36.0)	0.005

*Median (min–max) for continuous variables. p < 0.05 was considered statistically significant. PIVKA, protein induced by vitamin K absence or antagonist-II; TBIL, total bilirubin; DBIL, direct bilirubin; IBIL, indirect bilirubin; TBA, total bile acid; ALT, alanine aminotransferase; AST, aspartate transaminase; GGT, γ-glutamyl transpeptidase; TBA, total bile acid; AFP, Alpha-fetoprotein*.

### Changes of PIVKA-II and Clinical Biochemical Indicators During Hospitalization

During hospitalization, the serological indicators and PIVKA-II levels of the patients were monitored. As the disease gradually improved, the trend in the PIVKA-II and TB levels was similar; that is, both had peaks that appeared in the third week, which was 2 weeks later than that of transaminase. The peak of AFP appeared was 1 week later than that of PIVKA-II in the fourth week is shown in Table 3.

**Table 3:** Changes in PIVKA-II and clinical biochemical indicators during hospitalization.

**Time (weeks)**	** <1**	**1–2**	**2–3**	**3–4**	**> 4**
PIVKA-II (mAu/mL)	45.8 (38.7–369.8)	74.7 (31.7–534.7)	1,194.3 (616.6–2,622.1)	41.1 (20.0–885.3)	36.2 (29.2–116.0)
TBIL (μmol/L)	135.3 (7.0–460.3)	160.2 (16.1–242)	245.3 (160–357)	245.0 (41.7–425)	37.8 (21–50.7)
DBIL (μmol/L)	107.8 (2.0–396.0)	130.2 (7–223)	216.5 (141–318)	212.3 (23.7–363)	21.8 (10.7–31.1)
IBIL (μmol/L)	27.5 (2–164.7)	12.4 (4–19)	28.8 (19–81)	32.8 (11–62)	15.9 (10.3–19.6)
ALP (U/L)	232.8 (61–475)	104.5 (95–123)	141.3 (120–166)	117.0 (109–143)	123.0 (95–131)
ALT (U/L)	798 (243.5–1,542.3)	89.0 (55.5–134.5)	87.0 (43.1–241.0)	33.0 (28.5–88.5)	21.5 (20.0–35.5)
AST (U/L)	296.0 (77.0–878.0)	42.0 (33.0–71.0)	89.0 (59.0–16.0)	41.0 (30.5–65.0)	41.0 (34.0–55.0)
GGT (U/L)	150.0 (91.3–443.3)	104.0 (72.0–160.5)	66.5 (51.7–88.5)	71.0 (66.5–143.5)	31.5 (21.0–45.5)
TBA (μmol/L)	210.4 (2–477)	142.6 (12.1–273)	239.7 (177.4–363)	41.7 (41.7–41.7)	8.8 (4.5–13.2)
AFP (ng/ml)	7.1 (1.8–27.8)	6.5 (4.1–1,032.1)	132.6 (41.8–396.4)	148.6 (8.1–119.1)	88.7 (7.8–98.0)
PT (s)	15.7 (1.0–337)	14.1 (13.5–14.9)	13.8 (13.4–14.3)	17.1 (13.1–20.7)	13.1 (13.1–13.1)
Albumin (g/L)	34.4 (31.9–38.5)	33.3 (28.9–36.3)	30.8 (30.3–34.0)	36.0 (34.3–41.1)	36.8 (36.5–38.4)

*TBIL, total bilirubin; DBIL, direct bilirubin; IBIL, indirect bilirubin; TBA, total bile acid; ALT, alanine aminotransferase; AST, aspartate transaminase; GGT, γ-glutamyl transpeptidase; TBA, total bile acid; AFP, Alpha-fetoprotein*.

### The Correlation Between PIVKA-II With Bilirubin and Albumin Levels

**Figure 3:**
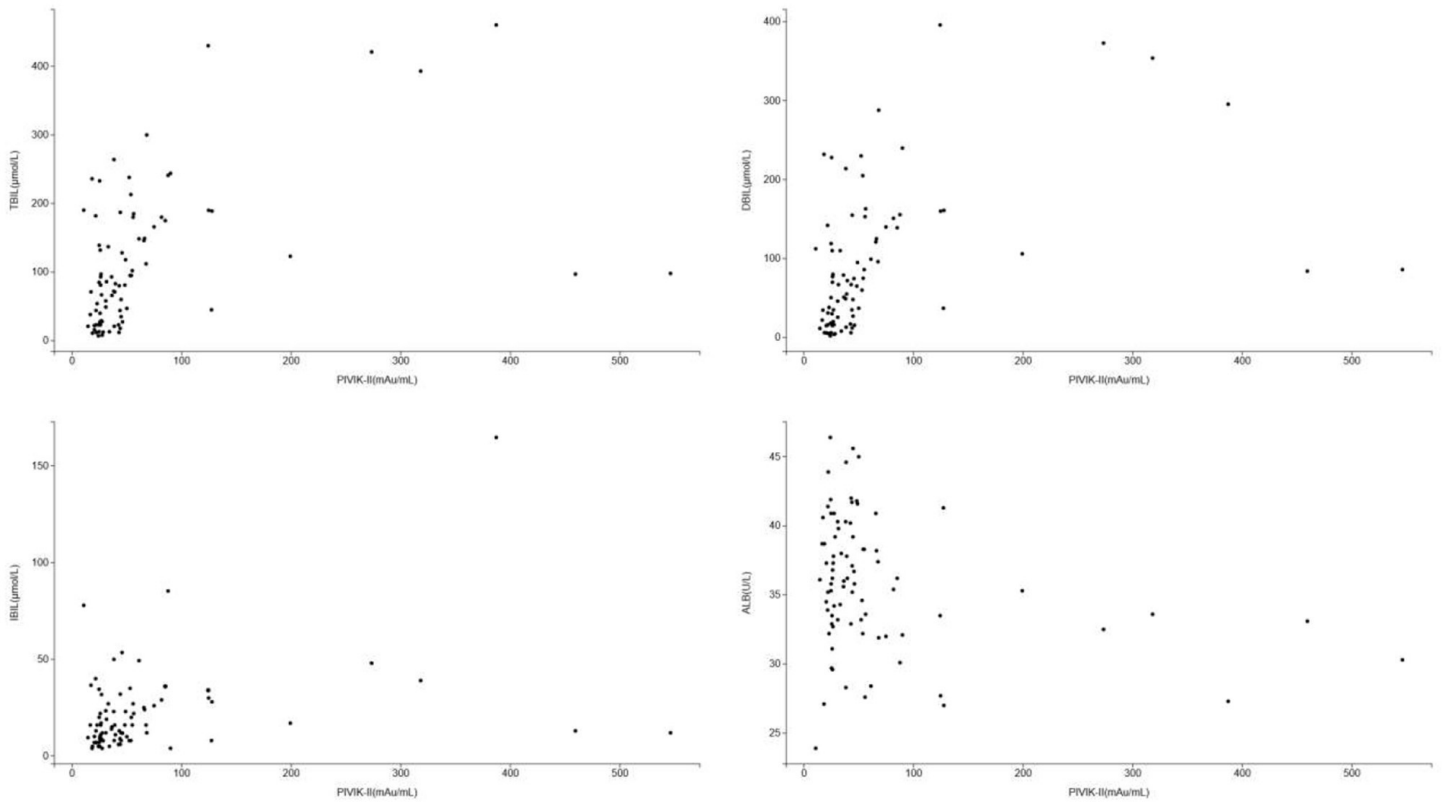
Scatter diagram for the correlation between PIVKA-II and bilirubin and albumin. Each black point represents a sample. PIVKA, protein induced by vitamin K absence or antagonist-II. PIVKA, protein induced by vitamin K absence or antagonist-II.

**Figure 4:**
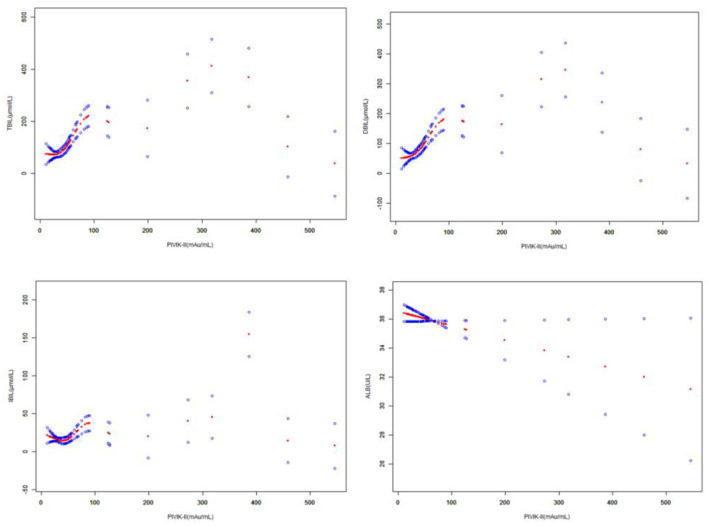
The correlation between PIVKA-II and bilirubin and albumin. The solid red line represents the smooth curve fit between variables. Blue bands represent the 95% CI from the fit. Gender and age were adjusted. PIVKA, protein induced by vitamin K absence or antagonist-II.

The scatter diagram and smooth curve fittings used to characterize the non-linear relationship between PIVKA-II with TBIL, DBIL, IBIL, and albumin levels are shown in Figures 3, 4. PIVKA-II is positively correlated with total bilirubin (*r* = 0.563, *p* = 0.00), positively correlated with direct bilirubin (*r* = 0.556, *p* = 0.00), positively correlated with indirect bilirubin (*r* = 0.357, *p* = 0.00), and negatively correlated with albumin (*r* = −0.264, *p* = 0.006).

The association between PIVKA-II with TBIL and DBIL was an inverted U-shaped curve, with the point of inflection identified using a two piecewise linear regression model, at 199.1 mAU/ml (Table 4). For a PIVKA-II <199.1 mAU/ml, every 1 mAU/ml increase in PIVKA-II was associated with a 1.6 μmol/L greater TBIL (95% CI: 1.1–2.0); by comparison, for individuals with a PIVKA-II >199.1 mAU/ml, a 1 mAU/ml increase in PIVKA-II was associated with a 0.7 μmol/L decrease in TBIL (95% CI: −1.1 to −0.2). Similarly, for a PIVKA-II <199.1 mAU/ml, every 1 mAU/ml increase in PIVKA-II was associated with a 1.4 μmol/L greater DBIL (95% CI: 1.0–1.8); by comparison, for individuals with a PIVKA-II >199.1 mAU/ml, a 1 mAU/ml increase in PIVKA-II was associated with a 0.6 μmol/L decrease in DBIL (95% CI: −1.0 to −0.3).

**Table 4:** Threshold effect analysis of PIVKA-II on TBIL, DBIL, IBIL, and albumin using the two-piecewise linear regression model.

**Parameters**	**Adjustedβ (95% CI), *P*-value**
TBIL	
Fitting by the standard linear model	0.4 (0.2, 0.6), <0.001
Fitting by the two-piecewise linear model	
Inflection point	**199.1**
PIVKA-II <199.1 (mAU/mL)	1.6 (1.1, 2.0), <0.001
PIVKA-II >199.1 (mAU/mL)	−0.7 (−1.1, −0.2), 0.003
Log likelihood ratio	<0.001
DBIL	
Fitting by the standard linear model	0.3 (0.1, 0.5) 0.002
Fitting by the two-piecewise linear model	
Inflection point	**199.1**
PIVKA-II <199.1 (mAU/mL)	1.4 (1.0, 1.8) <0.001
PIVKA-II >199.1 (mAU/mL)	−0.6 (−1.0, −0.3) 0.001
Log likelihood ratio	<0.001
IBIL	
Fitting by the standard linear model	(0.0, 0.1) 0.006
Fitting by the two-piecewise linear model	
Inflection point	**18.6**
PIVKA-II <18.6 (mAU/mL) PIVKA-II>18.6 (mAU/mL) Log likelihood ratio	−5.4 (−9.8, −0.9) 0.020 0.1 (0.0, 0.1) 0.002 0.014
Albumin	
Fitting by the standard linear model	
Fitting by the two-piecewise linear model	−0.0 (−0.0, 0.0) 0.064
Inflection point	**18.6**
PIVKA-II <18.6 (mAU/mL)	1.1 (0.2, 2.0) 0.017
PIVKA-II >18.6 (mAU/mL) Log likelihood ratio	−0.0 (−0.0, −0.0) 0.026 0.013

*Gender and age were adjusted. PIVKA, protein induced by vitamin K absence or antagonist-II*.

The association between PIVKA-II with IBIL was a U-shaped curve, with the point of inflection identified using a two piecewise linear regression model, at 18.6 mAU/ml (Table 4). For a PIVKA-II <18.6 mAU/ml, every 1 mAU/ml increase in PIVKA-II was associated with a 5.4 μmol/L lower IBIL (95% CI: −9.8 to −0.9); by comparison, for individuals with a PIVKA-II >18.6 mAU/ml, a 1 mAU/ml increase in PIVKA-II was associated with a 0.1 μmol/L increase in IBIL (95% CI: 0.0–0.1). However, the association between PIVKA-II with albumin was an inverted U-shaped curve, with the point of inflection identified using a two piecewise linear regression model, at 18.6 mAU/ml (Table 4). For a PIVKA-II <18.6 mAU/ml, every 1 mAU/ml increase in PIVKA-II was associated with a 1.1 μmol/L greater albumin (95% CI: 0.2–2.0).

## Discussion

Hepatitis E is distributed worldwide and is prevalent in many developing countries in Africa and Asia (9). In fact, the WHO has considered hepatitis E as an important public health problem in developing countries (2). In the recent years, with the improvement of the living standards of the Chinese population, the hepatitis E epidemic has been more controlled than that before, but there are still sporadic cases, and small-scale hepatitis E outbreaks have been reported from time to time (10–12). In addition, the age of onset has become a new feature in the epidemiology of hepatitis E in the country. An epidemiological survey on hepatitis E in China showed that the number of reported cases aged 45–69 years accounted for more than half of all reported cases (10). Moreover, Li et al. reported that the prevalence of anti-HEV antibodies had increased with age (13). Most cases of hepatitis E are self-limiting, and progression to acute liver failure is rare. However, older and diabetic patients are more likely to develop acute liver failure and die when compared with younger and non-diabetic patients (14). Pregnant women, especially those in their last trimester, had a case fatality rate of more than 10% (15, 16). In our current study, these patients had a median value of 53 years old ranged from 25 to 77. There were no significant differences in age distribution between the normal PIVKA-II group and the elevated PIVKA-II group. Pregnant patients were not included in our study and none of the 84 patients progressed to acute liver failure during hospitalization.

Protein induced by vitamin K absence or antagonist-II -II is a protein produced due to vitamin K deficiency or antagonists and is mainly composed of liver cell particles. Carboxylase system and epoxide reductase work together to produce PIVKA-II. The glutamic acid residues in the γ-carboxyglutamate structure are not fully carboxylated to γ-carboxyglutamate and lose their normal activity (17, 18). PIVKA-II has been recently found to have a good diagnostic value for early liver tumors, especially HCC (19). However, PIVKA-II elevation is not specific for HCC diagnosis, reported in many other disease situations. Moreover, Wu et al. reported that PIVKA-II levels in non-cirrhotic chronic hepatitis B patients were higher than that in the healthy control group (20). Other factors, such as vitamin K deficiency, taking warfarin, primary gastric adenocarcinoma, transplant rejection, lack of nutrition, intestinal flora imbalance, renal failure, inflammatory bowel disease, and alcoholic liver disease, led to increased serum PIVKA-II levels in non-HCC patients (6). Besides, Takikawa reported that PIVKA-II helps to monitor the severity of acute liver injury (21). In this study, we included 84 cases of patients with hepatitis E and measured the levels of the PIVKA-II and various serological indicators, and monitored the indicators during hospitalization. The PIVKA-II levels increased in 59.51% (50/84) of patients with hepatitis E, four cases had PIVKA-II levels >1,000 mAU/ml, the highest of which was 9,235 mAU/ml. We classified the degree of increase in the PIVKA-II levels into two groups. Compared with the slightly elevated PIVKA-II group, patients in the significantly elevated PIVKA-II group had much lower serum albumin, ALT, AST levels, and longer days for the hospital stay. An elevated PIVKA-II correlated with a greater TBIL, DBIL, IBIL, and lower albumin. Moreover, we identified a non-linear relationship between PIVKA-II with TBIL and DBIL with a point of inflection at 199.1 mAU/ml and a non-linear relationship between PIVKA-II with IBIL and albumin with a point of inflection at 18.6 mAU/ml. These results indicated that PIVKA-II levels might be related with the severity of hepatitis E.

An increase in prothrombin levels also leads to the production of abnormal prothrombin (18). Therefore, we believe that PIVKA-II may be associated with the severity of acute liver damage. Actually, the underlying mechanism behind the effect of HEV infection on serum PIVKA-II levels remains unclear. We believe that this may be related to liver cell damage and metabolic disorders in patients with hepatitis E, which decreased the ability of liver cells to synthesize protein and may weaken the function of vitamin K-dependent shuttling enzymes, causing the metabolic utilization of vitamin K. Besides, elevated PIVKA-II levels in hepatitis E may also be related with Vitamin K absence. Regretfully, the relationship between serum vitamin K concentration and serum PIVKA-II levels was not explored in our current study because data on serum vitamin K levels are unavailable. Moreover, no related pieces of literature were previously published about the relationship between Vitamin K absence and HEV infection. Therefore, whether Vitamin K absence existed in patients with HEV infection was still unclear and the effect of HEV infection on serum PIVKA-II levels needs to be clarified in future research.

With the gradual recovery of patients with hepatitis E, PIVKA-II levels also gradually decreased, and the peak was delayed by 2 weeks compared with the peak of transaminase, which was roughly similar to that of bilirubin. The peak of AFP was 1 week later than that of PIVKA-II. Many studies believe that AFP exists in the cytoplasm of oval cells or hyperproliferative cells (22, 23), which indicates the proliferation of liver cells after injury, and the new liver cells may synthesize AFP briefly in the early stage. We believe that PIVKA-II is related to liver cell damage, so there may be a certain node in the abnormal metabolism and hyperplasia of liver cell necrosis, which is a turning point in the disease of the patients with hepatitis E. However, we did not have enough cases in this study, and the sample size needs to be further expanded.

Based on the above information, it could be seen that more than half of patients with hepatitis E had elevated PIVKA-II levels, which were all transiently elevated, and the patients gradually recovered after a few weeks. With the recovery of patients from hepatitis E, none of the cases continued to progress. Therefore, PIVKA-II could be a significant reference for the course of hepatitis E disease. Moreover, we can refer to the PIVKA-II level of patients with hepatitis E to assess the severity of the patient's condition and the trend of the outcome to assist in the diagnosis and treatment of the disease further. If the patient's PIVKA-II continues to rise, further examinations should be undertaken, and the possibility of HCC should be ruled out. The association between PIVKA-II with TBIL, DBIL, IBIL, and albumin was a U-shaped or inverted U-shaped curve with an obvious point of inflection. However, this study had some limitations. First, this was a retrospective study. The data collection time was relatively short. The study population was recruited from only two centers. We need to conduct more cases and perform long-term follow-up evaluations. Second, we excluded individuals with HCC from our study sample as HCC may have a significant influence on PIVKA-II. Third, there remains the possibility of bias caused by other potential confounding factors that we did not adjust for.

## Data Availability Statement

The research involves patient private information, the data that support the findings of this study are available from the corresponding author upon reasonable request.

## Ethics Statement

The studies involving human participants were reviewed and approved by the Ethics Committee of the First Affiliated Hospital of Wenzhou Medical University and Taizhou Hospital of Zhejiang Province (approval number: No. 2021-zz-162). Due to the retrospective study design involving electronic health records and no additional interventions, written informed consent was waived from the patients or their relatives.

## Author Contributions

YC, YY, NY, and MLu contributed to the conception and design of the study. YC organized the database. YY performed the statistical analysis. YC wrote the first draft of the manuscript. SL, MLi, XX, HS, YJ, SZ, and HS wrote sections of the manuscript. All the authors contributed to manuscript revision, read, and approved the submitted version.

## Conflict of Interest

The authors declare that the research was conducted in the absence of any commercial or financial relationships that could be construed as a potential conflict of interest.

## Publisher's Note

All claims expressed in this article are solely those of the authors and do not necessarily represent those of their affiliated organizations, or those of the publisher, the editors and the reviewers. Any product that may be evaluated in this article, or claim that may be made by its manufacturer, is not guaranteed or endorsed by the publisher.
